# Evaluating reach, adoption, implementation and maintenance of Internet-based interventions to prevent eating disorders in adolescents: a systematic review

**DOI:** 10.1093/eurpub/ckz130

**Published:** 2019-08-13

**Authors:** Michael Zeiler, Stefanie Kuso, Barbara Nacke, Lisa M Klesges, Karin Waldherr

**Affiliations:** 1 Eating Disorder Unit, Department for Child and Adolescent Psychiatry, Medical University of Vienna, Vienna, Austria; 2 FernFH Distance Learning University of Applied Sciences, Wr. Neustadt, Austria; 3 Technische Universität Dresden, Institut für Klinische Psychologie und Psychotherapie, Dresden, Germany; 4 School of Public Health, University of Memphis, Memphis, TN, USA

## Abstract

**Background:**

Past research has yielded promising results on the effectiveness of Internet-based interventions to prevent eating disorders (EDs) in adolescents, but further information is needed to evaluate the public health impact of their large-scale dissemination. This article used an established framework to systematically review the extent to which indicators of the reach, effectiveness, adoption, implementation and maintenance [cf. Reach-Effectiveness-Adoption-Implementation-Maintenance (RE-AIM)-framework] of universal and targeted online ED prevention programmes are reported in the literature, in order to estimate their future dissemination potential.

**Methods:**

The literature search was conducted on PubMed, Web of Science and PsycINFO, and complemented by searching existing reviews and the reference lists of the studies included. Twenty-two studies published between 2000 and April 2019 met the inclusion criteria. We extracted data on a total of 43 indicators, within RE-AIM dimensions for each article, including qualitative coding of fostering and hindering factors.

**Results:**

Reach (55.0%) and implementation (54.0%) were the dimensions reported on most frequently, followed by effectiveness (46.8%), adoption (34.7%) and maintenance (18.2%). While internal validity indicators were frequently reported (e.g. sample size, effects and intervention intensity), most studies failed to report on elements of external validity, such as representativeness of participants and settings, adoption rates, implementation costs and programme sustainability.

**Conclusions:**

Evidence indicates that Internet-based ED prevention programmes can reach a large number of adolescents and can be feasibly implemented in school settings. However, given the paucity of large-scale dissemination studies available for review, the degree to which schools are willing to adopt preventive interventions, as well as the transferability of programmes to different settings and geographical regions remains unclear.

## Introduction

Eating disorders (EDs) risk is commonly reported among adolescents in Western societies.^1^ Unhealthy dieting or fasting practices, the over-estimation of shape and weight, body dissatisfaction, a drive for thinness and binge eating behaviour are common among adolescents,^2–4^ tend to remain stable or increase over time^5^^,^^6^ and may develop into a clinical ED.^7^ Thus, providing prevention and early intervention in this field is of high public health relevance. Internet-based interventions provide a promising public health approach meeting this challenge as they are generally being easy to disseminate, cost-effective, especially appropriate for adolescent users and requiring low staff support, and they can be tailored to individual needs and risks.^8^^,^^9^ In the last two decades, several Internet-based programmes for preventing EDs in adolescents have been developed, covering the spectrum from universal to selected and indicated prevention. ‘StudentBodies’, and its adaptation ‘Staying Fit’, offer the most comprehensively evaluated Internet-based prevention programme for EDs to date. In adolescent US samples, ‘StudentBodies/StayingFit’ have been found to be effective in reducing restricted eating and increasing knowledge about EDs in female 10th graders,^10^ reducing weight and shape concerns and binge eating among male and female high-school students with overweight,^11^ and reducing body dissatisfaction and increasing fruit and vegetable consumption in 9th grade students of normal weight or overweight.^12^^,^^13^ ‘ProYouth’, an open-access Internet-based ED prevention programme in Europe, and its pre-version ‘YoungES[S]PRIT’, showed variable effectiveness in decreasing the incidence rate of clinical EDs compared with that of a control group^14^ but clear effectiveness in facilitating help-seeking behaviour.^15^

Recent meta-analyses confirm the effectiveness of Internet-based ED prevention programmes in adult and adolescent samples. Melioli et al.^16^ found small to medium effect sizes among 20 Internet-based programmes, successfully decreasing body dissatisfaction, internalization of the thin ideal, shape/weight concerns, dietary restriction, the drive for thinness, bulimic symptoms, purging frequency and negative affect and Loucas et al.^17^ observed small effects of online prevention programmes on ED psychopathology, weight concerns and drive for thinness. As concluded in the most recent meta-analysis by Le et al.,^18^ which included but was not restricted to Internet-based approaches, ED prevention programmes targeting high-risk adolescents were more effective than were universal prevention programmes.

While emerging evidence supports the efficacy of Internet-based ED prevention programmes in adolescents, little is known about their dissemination potential. Evaluation of the generalizability and the public health impact of ED prevention programmes would support future efforts for their wider dissemination. One such evaluation approach, the RE-AIM (Reach-Effectiveness-Adoption-Implementation-Maintenance) model,^19^ provides a useful framework for evaluating the real-world effectiveness and external validity of interventions and proposes standards in reporting on behavioural interventions. RE-AIM has been successfully used in other systematic reviews evaluating the public health impact of interventions targeting, e.g. physical activity,^20^^,^^21^ childhood obesity,^22^^,^^23^ dietary intake,^24^ diabetes self-management,^25^ health literacy^26^ and mood disorders.^27^

The purpose of this article was to conduct a systematic literature review using the RE-AIM framework to provide a comprehensive evaluation of the external validity and dissemination potential of Internet-based ED preventive interventions targeting adolescents. We aimed to analyse reporting rates for each RE-AIM dimension, as well as indicators of individual participant reach, organizational adoption, implementation fidelity and maintenance at the individual and setting level. We anticipate that the results of this systematic review will identify research gaps as well as provide insights into factors relevant to the recruitment of adolescents and organizations (e.g. schools) and the sustainable implementation of Internet-based ED prevention programmes for adolescents.

## Methods

### Study design

A systematic review was conducted using the RE-AIM framework.^19^ The framework covers five dimensions including ‘reach’ (ability to engage a high number of participants), ‘efficacy/effectiveness’ (effects of the intervention on health outcomes), ‘adoption’ (ability to engage a high number of settings/organizations who are willing to offer the intervention), ‘implementation’ (degree to which the intervention is delivered as intended) and ‘maintenance’ (including both, maintenance of effects at the individual level and sustainability of the intervention at an organizational level). The reporting of this study adheres to the PRISMA guidelines.^28^ No review protocol was published in advance.

### Information sources and search strategy

Literature published from 1 January 2000 until 9 April 2019 was included. The date range was based on previous reviews in this field that indicated the first Internet-based programmes were developed in the early 2000s. Three electronic databases (PubMed, PsycInfo and Web of Science) were searched. Selected keywords related to (prevention) programmes (program* OR *intervention* OR *application* OR *app* OR *self-help* OR prevent* OR *health promotion*), technology (*online* OR internet* OR computer* OR web* OR *e-mental health* OR *e-health* OR *ehealth* OR technol* OR *digital* OR *mobile* OR *smartphone* OR *tablet* OR *blended*) and to the topic of eating disorders (*eating disorder* OR *anorexia* OR *bulimia* OR *binge eating* OR *EDNOS* OR *OSFED* OR *disordered eating* OR *restrained eating* OR *eating pathology* OR chronic diet* OR *body dissatisfaction* OR *intuitive eating* OR *weight regulation* OR *body image* OR *eating behavior* OR *eating behaviour*). These three groups of keywords were connected with an ‘AND’-statement. As the literature search for this review was merged with another review focusing on adult programmes,^29^ it was not limited to children and adolescents at this stage. The exact search syntax is provided in Supplementary table S1.

Furthermore, 32 published narrative and systematic reviews focusing on ED intervention programmes, as well as reference lists from the included studies, were searched to augment the database search.

### Eligibility criteria

To be eligible, studies had to meet the following inclusion criteria: (i) it had been published in a peer-reviewed journal; (ii) its publication language was English or German; (iii) the original study type was longitudinal and assessed Internet-based interventions; however, cross-sectional studies were also included if at least one RE-AIM dimension was reported in the article (e.g. reach of an Internet-based programme); (iv) the prevention programme included universal, selected or indicated prevention–we excluded clinical treatment or case series reports of a fully diagnosed ED (i.e. not a universal, selected or indicated prevention programme) or population-based studies that included more than 50% of the sample with full-syndrome EDs; (v) the programme aimed to prevent EDs or reduce risk factors for EDs indicated by at least one of the following: declared itself a prevention programme for EDs, aimed at reducing body image concerns or body dissatisfaction, or promoting balanced eating habits–e.g. a study was excluded if the main focus was to reduce weight or caloric intake or on specific healthy or unhealthy food intake; (vi) the programme was fully or partly technology-based and delivered via computer, tablet or smartphone–mixed interventions combining face-to-face and Internet technology were included; and (vii) the programme was mainly targeted at preventing EDs in children or adolescents (up to the age of 17 years)–programmes approaching parents or teachers were included.

### Study selection

All citations identified through the database search and additional sources were imported into a reference manager. Duplicate articles and non-journal citations (books, book chapters, theses and conference abstracts) were excluded. All remaining abstracts were screened for eligibility by two independent researchers. Full-texts of relevant articles were obtained and checked for inclusion criteria by at least two researchers independently (M.Z., S.K. and B.N.). Any disagreements were discussed with a senior researcher (K.W.) until consensus was reached.

### Data extraction and scoring

The data extraction from articles meeting the inclusion and exclusion criteria was based on a previously validated coding sheet for conducting RE-AIM reviews published at www.re-aim.org.^30^^,^^31^ The coding sheet comprises a set of items (subsequently called indicators) that are, when reported in the literature, indicative for reach of participants, programme effectiveness, organizational adoption, implementation and sustainability of intervention programmes. The coding sheet was adapted to suit Internet-based interventions by revising the adoption elements. The reason for this was that web-based interventions may not be settings-based, nor require a delivery agent. Furthermore, for implementation we coded the intervention format (either web-based, computer-based only or mixed), the level and type of staff needed to support the intervention delivery, the electronic devices used (e.g. computer, smartphone and tablet), any data protection measures described, and whether incentives were used for programme and assessment completion. Despite these additions, minimal changes were made to the original definitions on the RE-AIM coding sheet. All adaptations were discussed between an RE-AIM expert (L.M.K.) and members of the ICare consortium who are experienced in Internet-based interventions. All the indicators that were extracted as well as the rationale for changes made to the original RE-AIM coding sheet are described in Supplementary table S2.

In total, 10 indicators were coded for the reach dimension, 7 for efficacy/effectiveness, 12 for adoption, 9 for implementation and 5 for maintenance. For each of these RE-AIM indicators the coders indicated whether or not the indicator had been reported on (yes vs. no) and if reported, specific data were extracted. The number and percentage of reported indicators for each RE-AIM dimension was calculated separately for each study included in the review. Summative reporting rates were calculated for each RE-AIM indicator by dividing the number of studies reporting on an indicator by the total number of studies reviewed. All studies were coded independently by two researchers (M.Z. and S.K. or B.N.). Across all studies and RE-AIM indicators the percentage of matching codes (reported vs. not reported) was 89.3% (Cohens’ Kappa: 0.78), indicating good inter-rater reliability. Inconsistencies and difficulties were discussed with a third independent researcher (K.W. or L.M.K.) until consensus was reached. In addition, two researchers (M.Z. and S.K.) independently extracted fostering and hindering factors for reach, adoption, implementation and maintenance when mentioned in the articles. These factors were categorised into meaningful themes using a thematic analysis approach.^32^ Consensus was reached through discussion between the coders.

## Results

### Study selection

**Figure 1:**
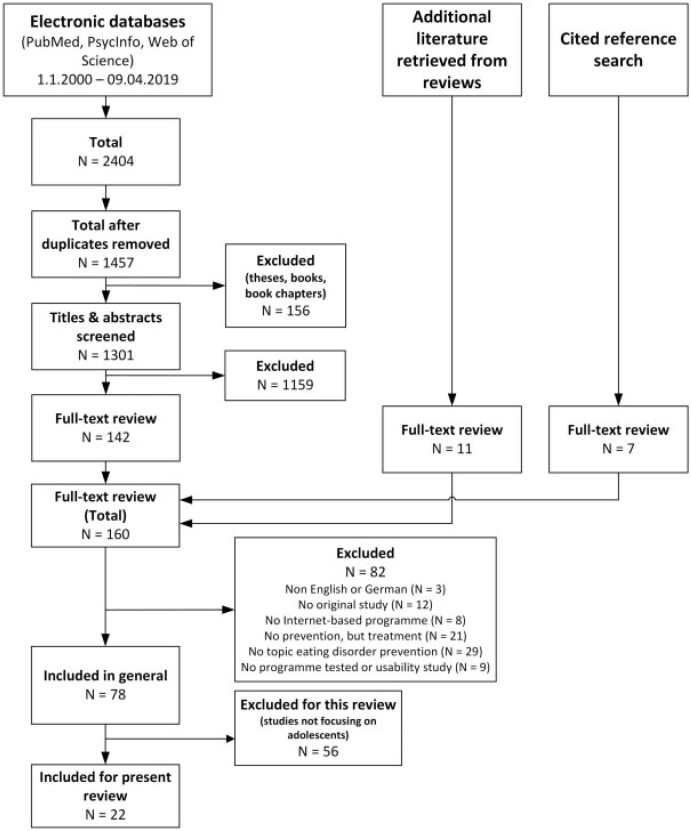
Flow diagram of studies included in the review

Figure 1 presents the flow of studies included in this review. The database search returned 928 records from PubMed, 671 records from PsycInfo and 805 records from Web of Science (total: 2404 records). After removing duplicates and excluding non-journal articles 1301 records remained, of which the title and abstract were screened. Of those, 142 were retrieved for full-text review. An additional of 11 manuscripts were retrieved from published reviews and 7 full texts by screening the reference lists of the included studies. A total of 82 studies were excluded for not meeting the inclusion criteria on publication language, study type and online prevention programme for EDs. A further 56 studies were excluded because they did not focus on adolescents, leaving 22 studies included in the present review.

### General study and intervention characteristics

A summary of the included studies is provided in table 1. The majority (86%) were conducted in the USA (*n* = 13) and Germany (*n* = 7). One study was cross-sectional, describing reach for an Internet-based programme only; 7 were uncontrolled, single-arm studies; 3 were randomized trials without a control group, testing the effects of different intervention groups; and 11 used a control group design, with 10 using a randomized control and 1 a quasi-experimental comparison group. Of the controlled trials, two used an active control group where participants received a control or placebo intervention; and nine used a treatment as usual, waiting list or non-active control group. Eight studies evaluated programmes in the ‘StudentBodies/StayingFit’ ‘family’ and five studies focused on ‘ProYouth’ or its earlier version ‘YoungES[S]PRIT’. Seven studies evaluated universal prevention programmes, four studies indicated prevention programmes and two studies selective prevention programmes, while nine combined universal with indicated/selective prevention. All the selective programmes were targeted at adolescents with overweight, except for one that was targeted at elite sports students. Twenty-three percent of studies enlisted parents, teachers or coaches of the targeted population, while the remainder directly approached the target group. None of the evaluated interventions was tailored to the individual risk of participants (e.g. individualized modules based on individual ED-risk profile). However, some studies varied programme content for groups considered to be at higher ED risk and by using gender-specific content. Adolescents participating in the ‘ProYouth’ programme were able to select modules based on their individual interests and needs.

**Table 1:** General characteristics of included studies and RE-AIM reporting rates

Study characteristics			Intervention characteristics					RE-AIM reporting rates (% of *n* indicators)^a^					
Reference	Country^b^	Study type/arms^c^	Programme name	Level of prevention^d^	Theory^e^	Tailored	Target group approach	R (%) (*n* = 10)	E (%) (*n* = 7)	A (%) (max. *n* = 12)	I (%) (*n* = 9)	M (%) (max. *n* = 5)	Total (%) (max. *n* = 43)
Abascal et al. (2004)^33^	USA	RT/3 intervention groups, uncontrolled	Student Bodies	UNI, IND	Psyedu	Segmented population	Directly	40	14	33	67	0	36
Bruning Brown et al. (2004)^10^	USA	Quasi-experimental/intervention group, TAU control group	Student Bodies	UNI	Psyedu, CBT	No	Directly + parents	40	43	33	67	20	43
Celio Doyle et al. (2008)^34^	USA	RCT/intervention group, TAU control group	Student Bodies 2	SEL	CBT	Segmented population	Directly	80	86	33	78	0	60
Cousineau et al. (2010)^35^	USA	RCT/intervention group, active control group	Trouble on the tightrope	UNI	Psyedu	Segmented population	Directly	50	57	22	67	0	43
Franko et al. (2013)^36^	USA	RCT/intervention group, TAU control group	BodiMojo	UNI	SCT, other	Segmented population	Directly	50	71	33	67	0	48
Heinicke et al. (2007)^37^	AUS	RCT/intervention group, waitlist control group	MyBody MyLife	IND	CBT	No	Directly	70	71	42	56	40	56
Jacobi et al. (2018)^38^	DE	RCT/intervention group, waitlist control group	Eltern als Therapeuten (E@T)	IND	FBT	No	Parents	90	71	50	44	60	64
Jones et al. (2008)^11^	USA	RCT/intervention group, waitlist control group	Student Bodies 2-BED	IND	CBT	No	Directly	80	71	33	67	40	60
Jones et al. (2012)^39^	USA, DE	Single-arm/intervention group, uncontrolled	Parents Act Now	IND	FBT	No	Parents	70	43	33	56	0	45
Jones et al. (2014)^13^	USA	Single-arm/intervention group (two tracks), uncontrolled	Staying Fit	UNI, SEL	CBT	Segmented population	Directly	40	71	44	67	25	51
Kindermann et al. (2017)^40^^,^^f^	DE	Cross-sectional/intervention group (reach)	ProYouth	IND, UNI	Psyedu	Self-choosing modules	Directly	50	0	8	44	0	24
Lindenberg and Kordy (2015)^14^	DE	RCT/intervention group, active control group	Young ES[S]PRIT	IND, UNI^g^	Self-management	No	Directly	70	86	33	67	60	61
Luce et al. (2005)^41^	USA	Single-arm/intervention group (two tracks), uncontrolled	Student Bodies	UNI, IND	CBT	Segmented population	Directly	50	29	33	11	0	28
Martinsen et al. (2014)^42^	NOR	Cluster RCT/intervention group, non-active control group	Health, body and sport performance intervention programme	SEL	SCT, DBT	No	Directly + parents, coaches	70	57	42	44	40	51
McVey et al. (2009)^43^	CAN	RCT/intervention group, non-active control group	The Student Body	UNI	Psyedu	No	Teachers, health practitioners	40	43	17	67	0	36
Minarik et al. (2013)^44^^,^^f^	DE	Single-arm/intervention group, uncontrolled	ProYouth	IND, UNI^g^	Psyedu	Self-choosing modules	Directly	60	29	50	56	50	50
Moessner et al. (2016)^45^^,^^f^	DE	RT/intervention group (testing different dissemination strategies)	ProYouth	IND, UNI^g^	Psyedu	Self-choosing modules	Directly	50	0	78	11	0	33
Moessner et al. (2016)^15^^,^^f^	DE	Single-arm/intervention group, uncontrolled	ProYouth	IND, UNI^g^	Psyedu	Self-choosing modules	Directly	50	14	25	44	0	31
Rodgers et al. (2018)^46^	USA	RCT/intervention group, non-active control group	BodiMojo	UNI	Self-compassion	No	Directly	30	43	33	67	0	39
Taylor et al. (2012)^12^	USA	Single-arm/intervention group (two tracks), uncontrolled	Staying Fit	UNI, SEL	CBT, Psyedu	Segmented population	Directly	40	43	42	44	25	41
Watt et al. (2005)^47^	USA	Single-arm/intervention group, uncontrolled	Trouble on the tightrope	UNI	Self-esteem education	No	Directly	30	14	11	33	0	21
Whittemore et al. (2013)^48^	USA	Cluster RT/2 intervention groups, uncontrolled	HEALTH[e] TEEN	UNI	SLT	No	Directly	60	71	22	67	40	53
					**Average reporting rates across studies**			**55**	**47**	**35**	**54**	**18**	**44**

a R: reach; E: efficacy/effectiveness; A: adoption; I: implementation; M: maintenance.

b AUS: Australia; CAN: Canada; DE: Germany; NOR: Norway; USA: United States of America.

c RT: randomized trial; RCT: randomized controlled trial; TAU: treatment as usual.

d UNI: universal; SEL: selected; IND: indicated.

e CBT: cognitive behavioural therapy; DBT: dissonance-based theory; Psyedu: psychoeducational; SLT: social learning theory; SCT: social cognitive theory.

f The samples of these studies partly overlap. However, as they are not based on the exact same sample, they are treated as single studies.

g The intervention is designed as indicated prevention, actually. However, adolescents with no indication (eating disorder risk) can participate as well.

### RE-AIM dimensions

The percentage of RE-AIM indicators reported on per study is presented in table 1. Across all the studies, average reporting rates (% of required indicators) were highest for reach (55.0%) and implementation (54.0%) followed by efficacy/effectiveness (46.8%) and lowest for adoption (34.7%) and maintenance (18.2%). The reporting rates for each single RE-AIM indicator across studies are presented in table 2 and the reporting status for each indicator per study is provided in Supplementary table S3.

**Table 2:** Reporting rates for RE-AIM indicators across included studies (*N* = 22)

RE-AIM indicator	Reporting rate (%)	RE-AIM indicator	Reporting rate (%)
Reach (total)	55.0	A5. Characteristics of approached setting	54.5
R1. Method to identify target population	54.5	A6. Characteristics of non-approached settings	0.0
R2. Inclusion/exclusion criteria	72.7	A7. Representativeness of participating settings	0.0
R3. Exclusion rate	36.4	A8. Reasons for declining of settings	4.5
R4. Sample size	100	A9. Method to identify delivery agent (*n* = 12)	8.3
R5. Participation rate	68.2	A10. Description of staff delivering intervention (*n* = 12)	58.3
R6. Characteristics of participants	90.9	A11. Level of expertise of delivery agent (*n* = 12)	50.0
R7. Characteristics of non-participants	0.0	A12. Start-up costs	4.5
R8. Representativeness of participants	9.1	Implementation (total)	54.0
R9. Reasons for declining participation	22.7	I1. Format of intervention	100
R10. Recruitment strategies	95.5	I2. Frequency and intensity of intervention	90.9
Efficacy/effectiveness (total)	46.8	I3. Level/type of staff support needed	86.4
E1. Measures and results for post-intervention assessment	86.4	I4. Electronic devices used	54.5
E2. Intention-to-treat analysis utilized	40.9	I5. Extent to which intervention was delivered as intended	59.1
E3. Imputation procedure	22.7	I6. Consistency of intervention delivery	13.6
E4. Quality of life measure included	0.0	I7. Costs of delivery	4.5
E5. Measure of satisfaction with/acceptability of programme	54.5	I8. Incentives used	40.9
E6. Effects at follow-up	50.0	I9. Data protection measures	36.4
E7. Attrition	72.7	Maintenance (total)	18.2
Adoption (total)	34.7	M1. Assessed outcomes ≥6 months	31.8
A1. Type(s) of included settings	100	M2. Drop-out rate to last follow-up (*n* = 7)	85.7
A2. Geographical characteristics of setting	81.8	M3. Current status of programme	22.7
A3. Inclusion and exclusion criteria for settings	13.6	M4. Adaptations made	0.0
A4. Adoption rate	40.9	M5. Costs of maintenance	4.6

#### Reach

A total of 72.7% of studies reported ‘inclusion and/or exclusion criteria’, in part reflecting the targeted prevention approaches. Inclusion criteria included specifying a gender or age range of participants, overweight status, self-reported body image concerns or specific ED symptoms, parental consent and access to the Internet. Exclusion criteria mainly referred to full-syndrome EDs, current ED treatment, medications use, medical conditions and low cognitive functioning. Studies reporting no inclusion/exclusion criteria mainly used universal approaches. More than half of the studies (54.5%) used screening assessments (either in-person, online or via telephone) to ‘identify the target population’. ‘Exclusion rates’ (reported by 36.4% of studies) ranged between 0.3% and 89% (median: 9.5%). All studies reported a ‘sample size’ defined as the number of students, parents or teachers who registered for or consented to participate in the programme. Of studies that directly recruited adolescents (*n* = 19), sample size ranged from 32 to 1667 (median: 274; 25th quantile: 83; 75th quantile: 455). Studies that recruited parents to reach adolescents reached 46–69 participants, and in one study, 78 elementary school teachers and 89 local health practitioners were reached.^43^

Two-thirds of studies reported data that allowed a ‘participation rate’ to be calculated. Eight studies contrasted the number of adolescent participants with the number of students in the sampled population. In seven of those studies,^10^^,^^12^^,^^14^^,^^33^^,^^35^^,^^36^^,^^48^ participation rates ranged between 39.5% and 97% (median: 69%). Moessner et al.^45^ reported both the total number of pupils in a geographical region in Germany (*N* = 288 507) and the number of registrations for the programme after disseminating information to schools in the area (*N* = 455), revealing a total population participation rate of 0.16%. Data reported by four studies^11^^,^^34^^,^^41^^,^^42^ allowed the calculation of a participation rate based on the number of eligible adolescents (range: 22–89%), while another^44^ reported the percentage of registrations of those who had completed a preceding self-test (41%).

Almost all the studies (90.9%) provided some information about the ‘characteristics of participants’. Six studies included girls only,^10^^,^^33^^,^^37–39^^,^^41^ while the remaining studies had predominately female participants (range: 33.7–92.9%, median: 63.3%). Mean age was between 13 and 16 years in the majority of studies, although two studies^35^^,^^47^ included younger children and three^40^^,^^44^^,^^46^ additionally included young adults. The percentage of non-Caucasian participants (reported by 13 studies, from the USA, Canada and Australia) ranged between 0% and 69% (median: 43.6%). No information was provided by any study regarding the ‘characteristics of non-participating adolescents’. Two studies provided information on the ‘representativeness of participants’, while one compared the socioeconomic status of participants with population estimates from census data^37^ and another compared ED risk status between participants and non-participants.^38^ ‘Reasons for declining participation’ were also rarely reported (22.7%), and when reported, they were very non-specific (e.g. no interest). One exception was Jacobi et al.,^38^ who collected detailed reasons why parents of children with elevated ED risk did not participate in their indicated online prevention programme.

All studies used school settings for the ‘recruitment’ of individual participants. Where details on recruitment strategies were given (95.5%), (i) information material was most frequently distributed by teachers, school counsellors or student representatives; (ii) presentations and workshops were held in schools; or (iii) participants were recruited in the course of school-based screening. In addition to recruitment via schools six studies used other strategies, including local newspapers,^34^ online advertisements (e.g. link on websites and social media)^15^^,^^40^^,^^44^ and the distribution of information material via health care or youth organizations.^34^^,^^43^^,^^46^

Regarding fostering and hindering factors for reaching individual participants, specific recruitment strategies (via school counsellors,^37^ online advertisements and referrals^39^) were emphasised as being beneficial. Two studies reported that the majority of participants received recruitment information via schools compared with other means (Internet, peers and flyers).^15^^,^^44^ Moessner et al.^45^ compared different school-based strategies and found that more intensive recruitment strategies (presentation and workshops at school) resulted in more participants registering compared with less intensive strategies (e.g. distributing printed information materials only). Teacher enthusiasm and the training of school staff, provider availability in the geographical region and political support were also discussed as fostering factors,^39^^,^^48^ whereas participating in other programmes at the same time and low awareness of ED-relevant issues were discussed as hindering factors.^39^^,^^41^ Aspects of stigmatization were also discussed: first, in terms of greater willingness to participate among adolescents who experienced feelings of shame in face-to-face interventions^37^ and conversely, in terms of hindering when registration was recommended in feedback from an ED screening, which could be experienced as wrong or embarrassing.^41^ Finally, recruitment incentives were mentioned as being beneficial in increasing participation.^38^

#### Efficacy/effectiveness

The majority of studies (86.4%) reported on ‘post-intervention effects’ of the programme, with 40.9% using ‘intent-to-treat analyses’ and the remainder analysing only those completing the study. Of the uncontrolled studies, significant pre-post improvements for entire samples or subsamples were reported for ED symptoms,^33^^,^^41^ weight and shape concerns,^12^^,^^13^^,^^41^ body mass index (BMI),^12^^,^^13^ healthy or balanced dietary intake,^12^^,^^13^^,^^48^ physical activity or sedentary activities,^13^^,^^48^ self-efficacy^48^ and knowledge about EDs and puberty-associated body changes.^47^ Moessner et al.^15^ found that 9.5% of participants had gone on to help-seeking behaviour, 24.4% of whom attributed this to programme participation; while 41.1% reported they would use treatment if needed, more than 50% of whom attributed this to programme participation. Of the controlled studies, relative improvement following intervention compared with control groups was observed for ED symptoms,^10^^,^^11^^,^^14^^,^^34^^,^^37^^,^^42^ weight shape concerns or body image,^11^^,^^35–37^^,^^46^ BMI,^11^^,^^34^^,^^38^ knowledge about EDs and related risk factors,^10^^,^^43^ self-esteem,^35^ self-compassion^46^ and depression.^37^ No study reported on effects on ‘quality of life measures’. Details on the significance/non-significance of effects are provided in Supplementary table S4.

‘Short-term follow-up effects’ (<6 months) were analysed in six studies, five of which found the maintenance of at least one outcome measure.^34^^,^^35^^,^^37^^,^^46^^,^^48^ ‘Measures of programme satisfaction or acceptability’ were included in 54.5% of studies, mostly in the form of single questions about overall programme satisfaction, perceived helpfulness, ease of use, content comprehensiveness and enjoyment. ‘Attrition rates’ were reported by 72.7% of studies: four^11^^,^^14^^,^^37^^,^^44^ provided information about early drop-out (prior to the first session) or rates of non-use of the intervention programme, which ranged from 2% to 23.1%; two^37^^,^^42^ reported on the drop-out rate during the intervention period (13.9% and 18.7%, respectively); and non-completion of post-assessments (reported by 13 studies) ranged from 1% to 81% (median: 19%; 25th quantile: 5%; 75th quantile: 50%). Only five studies compared programme completers and those who dropped out on sociodemographic characteristics, with three of these studies reporting no differences^37^^,^^43^^,^^46^ and two reporting significant differences in ethnic groups, BMI and degree of mental health problems.^11^^,^^42^

#### Adoption

All the studies approached schools to conduct individual recruitment, with four adding other ‘settings’ for recruitment including medical facilities,^34^^,^^39^ weight loss organizations,^34^ youth organizations^46^ and public health agencies.^43^ About half (45%) of schools also delivered the intervention in the school. Of studies that mentioned the number of settings, schools respectively, (*n* = 16), most included five or fewer settings (10 studies), while three included between 16 and 86 and two included 200 or more (specific to the e ‘ProYouth’ intervention^44^^,^^45^). Most studies were restricted to a specific region, and the majority gave a ‘description of the geographical area’ in which the programme was run (82%).

Only 14% of studies provided ‘setting-level inclusion/exclusion criteria’. General ‘description of setting characteristics’ (reporting rate: 55%) included information about public vs. private schools (*n* = 3 public, *n* = 1 private, *n* = 2 mixed), the distribution of school types (two studies^38^^,^^45^) and the ethnic diversity of the schools’ student population (two studies^12^^,^^13^). No study described the ‘characteristics of non-participating settings’ or assessed the ‘generalizability of settings’. However, 40.9% of studies reported the ‘participation rate of settings’ that were approached, this adoption rate ranging between 5.7% and 100%.

In 12 studies (55%) professional staff (mainly members of the study team with psychology training), delivered parts of the programme which consisted of moderating discussion groups or chat sessions, providing individual feedback and holding face-to-face sessions in blended interventions. Among these 12, 58% gave a ‘description of delivery staff’ as being primarily psychologists, supervised psychology students or therapists. No study reported any specialist ‘training’ regarding the delivery of Internet-based programmes.

Moessner et al.^45^ was the only study to report on ‘start-up costs’ in terms of the costs per registered participant of the various dissemination strategies. In their study, published in 2016, costs were estimated to range from €6.86 per registration when advertising materials were sent to schools, up to €431.10 when introductory school-based presentations and workshops were added to the strategy. Additionally, fostering factors for adoption were reported by Moessner et al.,^45^ who analysed differences in adoption rate as a function of various dissemination strategies. They found that the adoption rate was lower for more intensive strategies (introductory presentation + workshop at schools: 6.9%; introductory presentation only: 23.0%) than for less intensive strategies (via student representatives and peers: 50.0%; phone calls + printed advertisements: 88.7%). Elsewhere, Jacobi et al.^38^ reported that a letter of recommendation from school authorities and increasing awareness of the study through press releases also increased adoption rate.

#### Implementation

All the studies reported on the ‘format of the intervention’. The primary modality was web-based delivery, although two studies combined online and face-to-face sessions,^42^^,^^43^ one combined online sessions with phone calls^39^ and another used a CD-ROM-based intervention.^47^ Almost all (90.9%) studies provided indicators on the intended ‘intensity of the intervention’ with four studies^14^^,^^15^^,^^40^^,^^44^ (those evaluating the ‘ProYouth’ and ‘YoungES[S]PRIT’ interventions) explicitly stating that no instruction was provided on how often or how long the intervention should be used. For the remainder, the median duration of the programme was 7.5 weeks (25th quantile: 6 weeks; 75th quantile: 10 weeks) while the weekly input time ranged from 30 to 90 minutes. A discussion board was included in 14 studies, a monitoring function (e.g. food, physical activity log and emotion diaries) in 16 studies and the option of any kind of feedback in 11 studies (either automated or personalized via a coach or moderator). Over half (59.1%) of the studies reported on indicators of ‘participants’ adherence’ to the intervention, including general use of the programme and its components, the percentage of content viewed/completed, use of monitoring and discussion groups and the duration of programme use. An overview of these measures is provided in Supplementary table S5.

‘Level and type of staff’ needed to support the delivery of the intervention was reported in 86.4% of studies with one study stating that no staff were required.^46^ Staff tasks included moderating discussion groups,^10^^,^^33^^,^^39^ providing feedback,^34^^,^^38^^,^^39^^,^^42^ offering individual/group counselling in case of worsening symptoms,^14^^,^^15^^,^^37^^,^^40^^,^^44^ contacting teachers to increase participation,^11^^,^^48^ responding to questions, e.g. regarding technical difficulties^12^^,^^13^^,^^34^^,^^48^ and monitoring progress.^38^^,^^48^ Furthermore, staff were also required to present the programme in school classes,^13^ participate in face-to-face sessions in order to familiarise users with the programme and increase their adherence to it,^11^^,^^43^ personally attend classroom-sessions to ensure participation^35^^,^^36^ and hold lectures and seminars in class.^42^

Of the studies reporting on the ‘type of electronic devices used’ (54.4%), only one specified mobile technology (smartphones)^46^ while the others were delivered solely via (school) computers.

Only one study gave the ‘costs’ associated with programme implementation (ProYouth), which between 2011 and 2013^44^ were €15 per participant per year and included costs for the technical provider and staff. One study reported that the largest costs were for monitoring discussion groups,^12^ while another study stated that the intervention was inexpensive to deliver without describing any specific costs.^13^

In all, 40.9% of studies provided information about ‘incentives’ given to participants. These included monetary incentives for completing assessments, course credits, and reimbursements for schools.

Fostering and hindering factors reported for programme implementation were divided into three main themes. (i) Factors that might have fostered ‘individual adherence and compliance’ included reminders and incentives,^34^^,^^37^ feedback,^38^^,^^39^^,^^48^ customization of programmes,^39^ structured interventions,^11^ high teacher involvement,^48^ high ED risk to the child in a parent intervention^38^ and the use of social media and competitions,^13^ while hindering factors included missing gender sensibility,^44^ competing demands (e.g. time constraints),^11^^,^^33^^,^^42^ unanticipated closure of the school,^48^ and fears and reservations.^38^ (ii) ‘Embedding the programme in the school setting’: under this theme, implementing the programme instead of homework during school hours was regarded as beneficial.^48^ Furthermore, implementing it at the end of the school year was regarded as hindering due to exam stress.^37^ The involvement of teachers and school administrators in all decisions of programme implementation was also mentioned as a fostering factor.^48^ In another study, teachers argued that health professionals should conduct the programme in class rather than teachers. However, training teachers to deliver the programme could be regarded as professional development, which could foster commitment to its implementation.^43^ Furthermore, shaping the programme content to fit the learning goals set by the curriculum could facilitate implementation because there would be no ‘lost’ time for teachers^12^^,^^43^ and the content could be put into practice immediately (e.g. when implemented in physical activity classes).^13^ (iii) ‘Programme features’ that might have fostered implementation included the use of communication and interactive tools,^34^^,^^37^^,^^44^^,^^48^ short programme duration,^35^ anonymity and confidentiality,^37^^,^^44^ technical stability,^14^ usability,^35^ lack of costs^44^ and flexibility around time and place.^44^

‘Data protection measures’ were reported by 36.4% of studies. These were mostly limited to password-protected secured websites,^34^^,^^36^^,^^43^ anonymous user login,^10^ automatic logoff after a defined time of inactivity^34^ and separation of assessment and programme data.^46^ Jones et al.^11^^,^^13^^,^^39^ gave detailed information on data protection measures and additionally on protection of the server, data encryption and removal of data after study completion. They also informed users of the security risks associated with online programmes and instructed them not to give out any identifying information or share usernames and passwords.

#### Maintenance

Only seven studies (31.8%) reported ‘follow-up of participants outcomes ≥6 months’, the longest follow-up period reported being 12 months after baseline assessment. Five of the seven reported the long-term maintenance of effects for at least one outcome measure.^11^^,^^37^^,^^38^^,^^42^^,^^48^ Six studies reported a ‘drop-out rate’ of between 5% and 65.6%. Five studies (22.7%) reported on ‘programme sustainability’, two of those indicating that the schools were willing to offer the programme (StayingFit) to their students in subsequent years,^12^^,^^13^ one indicating that the researchers were not allowed to implement the programme as part of the curriculum in schools,^38^ one indicating that the programme would be continued and further developed in an European project^14^ and one describing the programme (ProYouth) as having been freely available since 2011.^44^

Regarding factors influencing the maintenance of individual outcomes, greater parental involvement,^11^^,^^34^ extended communication tools,^36^ better tailoring of programmes^12^^,^^13^ and use of mobile technology, virtual reality and gamification^12^ were discussed as fostering factors. Hindering factors included short programme duration.^36^ For better programme sustainability, high satisfaction among teachers,^13^ embedding the programme in the school curriculum,^36^ stable co-operation with schools and multiplicators/facilitators^44^ and constant/increased participation numbers^44^ were all mentioned.

## Discussion

This systematic review found that key elements for assessing the dissemination potential of Internet-based ED prevention programmes for adolescents are rarely included in the current literature. While reporting of internal validity indicators (e.g. sample size, effects and intervention intensity) was high, most studies failed to report on external validity elements such as the representativeness of participants and settings, adoption rates, implementation costs and programme sustainability.

Describing the reach and representativeness of samples recruited to ED prevention programmes is a key factor in informing future dissemination efforts. For funders and policy-makers to make decisions whether to scale-up preventive interventions, they need information about how well programmes are received by the target group(s) and their anticipated recruitment potential. Most of the studies examined here provided information on sample size, characteristics of the participating sample and inclusion/exclusion criteria. However, high exclusion rates were seen in some studies (up to 89%) that screened for gender and levels of ED risk. In future, given that Internet technology can simultaneously provide universal and targeted ED prevention programmes,^12^^,^^13^^,^^41^ individual exclusion rates could be reduced by using group settings (like school classes).

Although large variations in participation rates were observed between studies, the characteristics of non-participants, sample representativeness and reasons for non-participation were rarely reported. This hampers evaluation of generalizability of results and limits an understanding of contextual factors, characteristics of the underlying population and similarity of sample characteristics between studies. It remains largely unclear whether ED prevention programmes are able to reach adolescents who are most in need of prevention, as highlighted in other reviews of prevention programmes.^20^^,^^22^^,^^24^ The samples included in the studies reviewed were predominantly female, which could reflect the higher prevalence of ED risk in girls, girls agreement to participate, or studies that exclude boys. To increase reach across the adolescent population, ED prevention programmes might consider gender-specific content and designs to enhance reach among boys.^44^

Most studies reported on programme effectiveness, with outcome variables including ED symptoms, weight/shape concerns, body image and BMI. However, there is a paucity of data regarding effects on broader outcome measures, such as quality of life, which if examined could allow similar outcomes to be assessed across studies and potential negative effects to be explored.^19^ As reported by others,^49^ although attrition rates varied considerably between the studies they were generally rather high. Most studies did not report on whether attrition was related to key social or demographic factors, which could influence the generalizability of effective outcomes in future interventions. Some articles discussed factors that may have influenced attrition, including incentives, feedback, teacher involvement, gender sensibility, customization and time constraints, but did not investigate their influence on individual-, organizational- or intervention-level attrition systematically.

Setting-level adoption of programmes is an important factor to consider in the dissemination of ED prevention programmes. In the studies reviewed, all studies approached schools for the recruitment of participants and about half additionally for delivering the intervention, which is not surprising given that schools are regarded as a key setting for adolescent mental health interventions.^50^ Most studies approached a low number of schools, omitting to report on how they were selected from a potentially larger available sample. Thus, data on the extent of setting uptake is scarce. Furthermore, no study gave information on non-participating settings or the representativeness of settings. Thus, it remains unclear whether effects and implementation issues can be generalized to other schools or schools in different geographical regions. Future studies should target this research gap and investigate factors influencing the willingness of settings to participate in Internet-based ED prevention programmes in order to facilitate their widespread dissemination.

One study that systematically investigated adoption rate as a function of different recruitment strategies provides a useful exemplar of how recruitment yield and intervention effects combine to achieve programme effectiveness.^45^ In this study, a large number of schools of all types within a defined geographical region were willing to participate in a programme with less intensive recruitment strategies, i.e. tantamount to low effort on their part, but high adoption, indicating promising dissemination potential. On the other hand, participation of individuals was revealed to be lower following less intensive recruitment strategies, thereby decreasing the anticipated public health impact of Internet-based ED prevention programmes.

In planning the future implementation of programmes, detailed descriptions of the key aspects of the intervention are needed in order to better understand its dissemination potential. Most studies in this review reported on the format and intensity of interventions as well as the level of staff support needed; the majority reported that professional staff, mainly psychologists, psychotherapists or research assistants were involved in delivering specific parts of the programme. Programme delivery support included moderation of discussion groups, chat sessions and providing feedback.

In considering large-scale dissemination of interventions, implementation costs are crucial in decisions. The findings of this review reveal a paucity of data on the costs and cost-effectiveness of implementing Internet-based ED prevention programmes. Implementation costs were reported by just one study, conducted between 2011 and 2013, as being €15 per participant per year.^44^ Cost-effectiveness studies are important prerequisites for sustainability and large-scale dissemination of e-health interventions^51^ and we recommend improvements in the reporting of information on costs for staff and input time, programme set-up, provision of Internet technology, and implementation and maintenance of Internet-based ED prevention programmes.

Two further aspects to consider are the long-term maintenance of individuals’ behaviour and the setting-level sustainability of programmes. The maintenance was the least reported RE-AIM dimension, with less than half of studies reporting on any such indicators. The few studies that did suggest that the evidence for long-term maintenance of effects is promising, but improved reporting is needed to provide more generalized knowledge to evaluate programme impact. Similarly, information about the sustainability of programmes is generally scarce, with the exception of the ‘StudentBodies’^10^ programme (and its adaptations) which has been continuously adapted since early 2000 and offered by different research teams in the USA and Europe, and the ‘ProYouth’ programme^44^ which has been freely available since 2011. Several fostering factors for sustainably implementing Internet-based ED prevention programmes in school settings were discussed in the literature, including their fit with the schools’ curriculum and annual plan, close co-operation with school staff, support from mental health professionals and ongoing efforts to increase reach and participation.

Internet-based prevention programmes hold great promise in terms of future population impact, as highlighted in this systematic review. Several studies emphasised the importance of providing communication tools in order to foster individual adherence to Internet-based ED prevention programmes.^34^^,^^37^^,^^44^^,^^48^ Mobile apps for mental health interventions are regarded as particularly suitable for adolescents, being associated with high acceptability and user satisfaction.^52^ However, this review revealed that to date mobile technology is rarely used to deliver ED prevention programmes to adolescents, and thus presents an area for future research. Internet privacy and data protection measures were reported by fewer than 40% of studies. Dealing with confidentiality and privacy appears to be increasingly important for Internet-based programmes, as anonymity and data protection were seen as important prerequisites for participating in such programmes in a recent stakeholder survey that included adolescents.^53^ We recommend future studies to include a detailed statement on data protection and confidentiality with regard to Internet-based technology.

### Limitations

In addition to the strengths of this review, some limitations are noted. First, our conclusions are limited to the extent to which the studies reported on specific RE-AIM indicators. It is possible that the researchers collected data but either did not report on them or plan to report them in subsequent publications. Second, the available literature was dominated by two research groups: that from the Stanford University and Technical University of Dresden, who are responsible for the ‘StudentBodies’, StayingFit and ‘Parents Act Now’ programmes; and the group from the University of Heidelberg, who created the ‘ProYouth’ programme. Thus, in any summary measure of reporting rates, publications from these two research groups are disproportionately represented. Third, in this review we did not set out to evaluate the effect sizes of Internet-based ED prevention programmes for adolescents nor factors associated with effectiveness. The purpose was not to estimate a meta-effect but to evaluate whether the current literature allows the ability to estimate the generalizability and population impact of findings. While not the purpose of this review, methodological limitations including the small number and heterogeneity of included studies would not have supported a meta-analytic approach.

## Conclusion

Emerging evidence suggests that Internet-based interventions for the prevention of EDs in adolescents can reach a large number of adolescents, especially via the school setting, and can significantly reduce ED symptoms and body image concerns. This promising potential is hindered by a lack of large-scale dissemination studies and a lack of reported data to evaluate the degree to which current interventions could be disseminated to different settings and geographical regions. We have revealed gaps in the reporting of external validity indicators, including data on the representativeness of participants and settings. Furthermore, few studies provided information on implementation costs, despite this kind of information being key for decision-makers. We recommend that the reporting of RE-AIM indicators be improved in future studies, with special consideration given to factors relevant for the adoption of ED prevention programmes in different settings, and the cost-effectiveness and sustainability of such interventions.

## Supplementary data

Supplementary data are available at *EURPUB* online.

## Funding

This project has received funding from the European Union’s Horizon 2020 research and innovation programme under grant agreement No 634757.

*Conflicts of interest:* None declared.

**Key points**

- Schools are regarded as the most relevant setting for reaching adolescents through Internet-based eating disorder (ED) prevention programmes.
- Data on the representativeness of participants and settings, as well as on the sustainability and cost-effectiveness of programmes are scarce.
- In half of Internet-based ED preventive interventions, mental health professionals delivered specific parts of the programme. This contact with professionals appears to be beneficial for adherence.
- The fit of the programme with the curriculum and annual plan of schools, close co-operation with school staff, support from mental health professionals and ongoing efforts to increase reach and participation were discussed as the most relevant factors for fostering implementation in school settings.
- Reporting on data protection and privacy issues with respect to Internet technology is recommended for future studies.

## Supplementary Material

ckz130_Supplementary_Material
