# Erratum to: Evaluating reach, adoption, implementation and maintenance of internet-based interventions to prevent eating disorders in adolescents: A systematic review

**DOI:** 10.1093/eurpub/ckab109

**Published:** 2021-07-07

**Authors:** Michael Zeiler, Stefanie Kuso, Barbara Nacke, Lisa M Klesges, Karin Waldherr

**Affiliations:** 1 Eating Disorder Unit, Department for Child and Adolescent Psychiatry, Medical University of Vienna, Vienna, Austria; 2 FernFH Distance Learning University of Applied Sciences, Wr. Neustadt, Austria; 3 Technische Universität Dresden, Institut für Klinische Psychologie und Psychotherapie, Dresden, Germany; 4 School of Public Health, University of Memphis, Memphis, TN, USA

*European Journal of Public Health*, 2019; doi:10.1093/eurpub/ckz130

This paper was intended for publication in the supplement ‘Volume 31 Supplement 1: E-Mental-Health: Exploring the evidence base and stakeholders' perspectives on internet-based interventions for the prevention of mental health conditions’ but was accidentally published in Volume 30 Issue 1. The paper has been taken out of the issue and republished in the supplement. The Publisher and Editor apologize for the error.

