# Supplementary material for: Evaluating reach, adoption, implementation and maintenance of Internet-based interventions to prevent eating disorders in adolescents: a systematic review
Source: Eur J Public Health. 2019 Aug 13;31(Suppl 1):i38–47. doi: 10.1093/eurpub/ckz130 (PMC8266527; doi:10.1093/eurpub/ckz130)
Supplement: ckz130_Supplementary_Material [file ckz130_supplementary_material.docx]

**Electronic supplementary material**

Supplement to:

**Evaluating Reach, Adoption, Implementation and Maintenance of internet-based interventions to prevent eating disorders in adolescents: A systematic review**

*European Journal of Public Health*

Michael Zeiler, Stefanie Kuso, Barbara Nacke, Lisa Klesges, Karin Waldherr

Mag. Michael Zeiler

Medical University of Vienna, Department for Child and Adolescent Psychiatry, Waehringer Guertel 18-20, 1090 Vienna, Austria

E-Mail: michael.zeiler@meduniwien.ac.at

**Table of content**

Table S1. Search syntax used for literature search in PubMed, PsycInfo and Web of Science …………. page 2

Table S2. RE-AIM coding sheet used for the present systematic review …….…………………………………… page 3

Table S3. Reporting rates for RE-AIM indicators across included studies ………………………………………… page 6

Table S4. Reporting of RE-AIM indicators for each included study ………………………………………………….. page 7

Table S5. Efficacy of Internet-based interventions for uncontrolled and controlled studies …………… page 9

Table S6. Adherence and compliance measures reported in included studies ……………………………….. page 12

References ……………………………………………………………………………………………………………………………………… page 14

**Table S1. Search syntax used for literature search in PubMed, PsycInfo and Web of Science**

| **PubMed** |
| --- |
| (online[Title/Abstract] OR internet*[Title/Abstract] OR computer*[Title/Abstract] OR web*[Title/Abstract] OR e-mental health[Title/Abstract] OR e-health[Title/Abstract] OR “ehealth”[Title/Abstract] OR technol*[Title/Abstract] OR digital[Title/Abstract] OR mobile[Title/Abstract] OR smartphone[Title/Abstract] OR tablet[Title/Abstract] OR blended[Title/Abstract])  AND  (program*[Title/Abstract] OR intervention[Title/Abstract] OR application[Title/Abstract] OR app[Title/Abstract] OR self-help[Title/Abstract] OR prevent*[Title/Abstract] OR health promotion[Title/Abstract])  AND  (eating disorder[Title/Abstract] OR anorexia[Title/Abstract] OR bulimia[Title/Abstract] OR binge eating[Title/Abstract] OR EDNOS[Title/Abstract] OR OSFED[Title/Abstract] OR disordered eating[Title/Abstract]OR restrained eating[Title/Abstract] OR eating pathology[Title/Abstract] OR chronic diet*[Title/Abstract] OR body dissatisfaction[Title/Abstract] OR intuitive eating[Title/Abstract] OR weight regulation[Title/Abstract] OR body image[Title/Abstract] OR eating behavior[Title/Abstract] OR eating behaviour[Title/Abstract]) |
| **Psyc Info** |
| *((online OR internet* OR computer* OR web* OR e-mental health OR e-health OR ehealth OR technol* OR digital OR mobile OR smartphone OR tablet OR blended)*  *AND*  *(program* OR intervention OR application OR app OR self-help OR prevent* OR health promotion)*  *AND*  *(eating disorder OR anorexia OR bulimia OR binge eating OR EDNOS OR OSFED OR disordered eating OR restrained eating OR eating pathology OR chronic diet* OR body dissatisfaction OR intuitive eating OR weight regulation OR body image OR eating behavior OR eating behaviour)).ti,ab.* |
| **Web of Science** |
| (TS=(online) OR TS=(internet*) OR TS=(computer*) OR TS=(web*) OR TS=(“e-mental health”) OR TS=(“e-health”) OR TS=(“ehealth”) OR TS=(technol*) OR TS=(digital) OR TS=(mobile) OR TS=(smartphone) OR TS=(tablet) OR TS=(blended))  AND  (TS=(program*) OR TS=(intervention) OR TS=(application) OR TS=(app) OR TS=(self-help) OR TS=(prevent*) OR TS=(“health promotion”))  AND  (TS=(“eating disorder”) OR TS=(anorexia) OR TS=(bulimia) OR TS=(“binge eating”) OR TS=(EDNOS) OR TS=(OSFED) OR TS=(“disordered eating”) OR TS=(“restrained eating”) OR TS=(“eating pathology”) OR TS=(“chronic diet*”) OR TS=(“body dissatisfaction”) OR TS=(“intuitive eating”) OR TS=(“weight regulation”) OR TS=(“body image”) OR TS=(“eating behavior”) OR TS=(“eating behaviour”)) |

**Table S2. RE-AIM coding sheet used for the present systematic review**

| **Item** | | **Description** |
| --- | --- | --- |
| **General study and intervention characteristics** | | |
| First Author and Publication Year |  | |
| Country / Countries of intervention delivery |  | |
| Level of prevention | universal, selected or indicated prevention^a^ | |
| Programme name |  | |
| Theoretical background of intervention | e.g. Cognitive behavioural therapy, psychoeducational | |
| Main aim of intervention | e.g. reducing weight / shape concerns | |
| Tailored programme (yes vs. no) | Tailoring means that the content of the programme is (partly) individualised to fit the user needs | |
| Target group approached directly or indirectly | directly: adolescents receiving a programme; indirectly: third party (e.g. parents, teachers) are approached to prevent EDs in entrusted adolescents | |
| Study arms | number and type of study arms and description of comparison group(s) | |
| Type of study | e.g. RCT, quasi-experimental study, uncontrolled pre-post study | |
| **REACH** |  | |
| R1. Method to identify target population | Process by which target population was identified for participation in the intervention | |
| R2. Inclusion / Exclusion criteria | Description of inclusion / exclusion criteria for individual participants | |
| R3. Exclusion rate | % of participants excluded by exclusion criteria | |
| R4. Sample size | Number of participants in study | |
| R5. Participation rate | % of individuals who participated in intervention (including denominator) | |
| R6. Characteristics of participants | % females, mean age, age range, other characteristics | |
| R7. Characteristics of non-participants | see above | |
| R8. Representativeness of participants | Comparison of characteristics between participants and non-participants | |
| R9. Reasons for declining participation | Description of reasons | |
| R10. Recruitment strategies | Strategies to recruit individual participants | |
| **EFFICACY / EFFECTIVENESS** |  | |
| E1. Measures and results for post-intervention assessment | Description of outcome measures | |
| E2. Intention-to-treat analysis utilised | Analysing participants in the groups in which they were randomised regardless of adherence | |
| E3. Imputation procedure | Description of imputation procedure used for Intention-to-treat analysis | |
| E4. Quality of Life measure included | Description of use and results for QoL | |
| E5. Measure of satisfaction with / acceptability of programme^b^ | Description of included measures regarding satisfaction and acceptability | |
| E6. Effects at follow-up | At least one follow-up assessment (after post-intervention assessment) included | |
| E7. Attrition | Description of type and % of attrition | |

| **Item** | **Description** |
| --- | --- |
| **ADOPTION** |  |
| Setting-based^c^ | Is the intervention setting based? (for recruitment only, for intervention delivery only, for recruitment and intervention delivery, not setting-based) |
| A1. Type(s) of included settings^d^ | Description of type of setting(s) (e.g. schools) |
| A2. Geographical characteristics of setting^d^ | Description of where the intervention was delivered |
| A3. Inclusion and exclusion criteria for settings^b^ | Description of eligibility criteria for settings |
| A4. Adoption rate^d^ | % approached of eligible settings, % of settings participated of approached settings |
| A5. Characteristics of approached setting^d^ | Description of main characteristics of approached settings |
| A6. Characteristics of non-approached settings^d^ | Description of main characteristics of settings not approached |
| A7. Representativeness of participating settings^d^ | Comparison of characteristics between approached and non-approached settings |
| A8. Reasons for declining of settings^d^ | Description of reasons |
| Delivery agent necessary to delivery intervention^e^ | yes, delivery agent necessary to deliver (parts of) the intervention; no, only gatekeeper necessary; no |
| A9. Method to identify delivery agent^f^ | Description of process by which staff was identified for delivery of intervention |
| A10. Description of staff delivering intervention^f^ | Characteristics of staff |
| A11. Level of expertise of delivery agent^f^ | Description of expertise (e.g. training) of delivery agents |
| A12. Start-up costs | Description of costs for intervention set-up |
| **IMPLEMENTATION** |  |
| I1. Format of intervention^g^ | e.g. web-based only, blended intervention |
| I2. Frequency and intensity of intervention | Description of intervention duration and frequency |
| I3. Level / Type of staff support needed^h^ | Description of tasks done by staff to deliver intervention |
| I4. Electronical devices used^i^ | Electronical devices used to complete programme |
| I5. Extent to which intervention was delivered as intended | Description of individual adherence, compliance, attendance and/or staff adherence |
| I6. Consistency of intervention delivery | Description of consistency across staff, time, settings and subgroups of participants |
| I7. Costs of delivery | Description of costs (time, money) |
| I8. Incentives used^j^ | Description of incentives for participation in study |
| I9. Data protection measures^k^ | Description of data protection measures that were implemented |
| **MAINTENANCE** |  |
| M1. Assessed outcomes ≥ 6 months | Follow-up periods ≥ 6 months post-intervention |
| M2. Drop-out rate to last follow-up^l^ | % drop-out (if follow-up assessment ≥ 6 months) |
| M3. Current status of programme | Description of current status after end of study |

| M4. Adaptations made | Description of adaptations made to foster sustainability of programme |
| --- | --- |
| M5. Costs of maintenance | Description of costs (money, time) related to sustainability of programme |

**Notes:**

^a^ We follow the terminology proposed by O’Connell et al.^1^: *Universal* preventive interventions are usually targeted to the general public or a whole population that has not been identified on the basis of individual eating disorder risk. *Selective* preventive interventions are targeted to individuals or a population subgroup whose risk of developing eating disorders is significantly higher than average. *Indicated* preventive interventions are targeted to high-risk individuals who are identified as having signs or symptoms foreshadowing an eating disorder but dot meet diagnostic levels at the current time.

^b^ This item was newly added as published guidelines for reporting Internet-based interventions recommend an extended evaluation of user acceptance and satisfaction.^2^

^c^ This item was newly added as not all Internet-based interventions may be implemented in a specific setting. This item is not used for calculating overall reporting rates.

^d^ coded only if the intervention was setting-based (either for recruitment, delivery or both); Explanation: As some online interventions are not meant to be implemented in a specific setting (e.g. online-only interventions) these setting-specific RE-AIM indicators are coded only if the intervention is setting-based. Otherwise, the resulting (overall) reporting rates would be biased.

^e^ This item was newly added as a delivery agent may not be needed for all Internet-based interventions.This item is not used for calculating overall reporting rates.

^f^  coded only if a delivery agent was needed; Explanation: As online interventions may be completely unguided and no delivery agent may be needed, RE-AIM indicators referring to delivery agents are coded only if a delivery agent is needed. Otherwise, the resulting (overall) reporting rates would be biased.

^g^ This item was newly added as the format of intervention (e.g. web-based only, blended) is an essential feature of Internet-based interventions which also may affect outcomes and attrition.^3^

^h^ This item was newly added as published guidelines for reporting Internet-based interventions recommend an extended reporting of the level of needed staff support.^2^

^i^ This item was newly added as compatibility with and use of mobile phones may affect adherence to Internet-based interventions. Thus, reporting of the electronical devices used to access Internet-based interventions should be reported.^4^

^j^ This item was newly added as many researcher provide incentives for participants of Internet-based interventions as adherence may be increased.^5^ Thus, reporting the incentives given is highly recommended to be reported in studies evaluating Internet-based interventions.

^k^ This item was newly added as published guidelines for reporting Internet-based interventions recommend an extended reporting of data protection measures.^2^

^l^ coded only if M1 = yes

**Table S3.** Reporting rates for RE-AIM indicators across included studies (N = 22)

| **RE-Aim Indicator** | **Reporting rate (%)** | **RE-Aim Indicator** | **Reporting rate (%)** |
| --- | --- | --- | --- |
| **REACH (Total)** | **55.0%** | A5. Characteristics of approached setting | 54.5% |
| R1. Method to identify target population | 54.5% | A6. Characteristics of non-approached settings | 0.0% |
| R2. Inclusion / Exclusion criteria | 72.7% | A7. Representativeness of participating settings | 0.0% |
| R3. Exclusion rate | 36.4% | A8. Reasons for declining of settings | 4.5% |
| R4. Sample size | 100% | A9. Method to identify delivery agent (n = 12) | 8.3% |
| R5. Participation rate | 68.2% | A10. Description of staff delivering intervention (n = 12) | 58.3% |
| R6. Characteristics of participants | 90.9% | A11. Level of expertise of delivery agent (n = 12) | 50.0% |
| R7. Characteristics of non-participants | 0.0% | A12. Start-up costs | 4.5% |
| R8. Representativeness of participants | 9.1% | **IMPLEMENTATION (Total)** | **54.0%** |
| R9. Reasons for declining participation | 22.7% | I1. Format of intervention | 100% |
| R10. Recruitment strategies | 95.5% | I2. Frequency and intensity of intervention | 90.9% |
| **EFFICACY / EFFECTIVENESS (Total)** | **46.8%** | I3. Level / Type of staff support needed | 86.4% |
| E1. Measures and results for post-intervention assessment | 86.4% | I4. Electronical devices used | 54.5% |
| E2. Intention-to-treat analysis utilised | 40.9% | I5. Extent to which intervention was delivered as intended | 59.1% |
| E3. Imputation procedure | 22.7% | I6. Consistency of intervention delivery | 13.6% |
| E4. Quality of Life measure included | 0.0% | I7. Costs of delivery | 4.5% |
| E5. Measure of satisfaction with / acceptability of programme | 54.5% | I8. Incentives used | 40.9% |
| E6. Effects at follow-up | 50.0% | I9. Data protection measures | 36.4% |
| E7. Attrition | 72.7% | **MAINTENANCE (Total)** | **18.2%** |
| **ADOPTION (Total)** | **34.7%** | M1. Assessed outcomes ≥ 6 months | 31.8% |
| A1. Type(s) of included settings | 100% | M2. Drop-out rate to last follow-up (n = 7) | 85.7% |
| A2. Geographical characteristics of setting | 81.8% | M3. Current status of programme | 22.7% |
| A3. Inclusion and exclusion criteria for settings | 13.6% | M4. Adaptations made | 0.0% |
| A4. Adoption rate | 40.9% | M5. Costs of maintenance | 4.6% |

**Table S4. Reporting of RE-AIM indicators for each included study**

| **Reference** | **REACH** | | | | | | | | | | **EFFICACY / EFFECTIVENESS** | | | | | | | **ADOPTION** | | | | | | | | | | | | **IMPLEMENTATION** | | | | | | | | | **MAINTENANCE** | | | | |
| --- | --- | --- | --- | --- | --- | --- | --- | --- | --- | --- | --- | --- | --- | --- | --- | --- | --- | --- | --- | --- | --- | --- | --- | --- | --- | --- | --- | --- | --- | --- | --- | --- | --- | --- | --- | --- | --- | --- | --- | --- | --- | --- | --- |
|  | R1 | R2 | R3 | R4 | R5 | R6 | R7 | R8 | R9 | R10 | E1 | E2 | E3 | E4 | E5 | E6 | E7 | A1 | A2 | A3 | A4 | A5 | A6 | A7 | A8 | A9 | A10 | A11 | A12 | I1 | I2 | I3 | I4 | I5 | I6 | I7 | I8 | I9 | M1 | M2 | M3 | M4 | M5 |
| Abascal et al. (2004)^6^ | - | - | - | + | + | + | - | - | - | + | + | - | - | - | - | - | - | + | + | - | - | + | - | - | - | n.a. | n.a. | n.a. | - | + | + | + | + | + | - | - | + | - | - | n.a. | - | - | - |
| Bruning Brown et al. (2004)^7^ | - | - | - | + | + | + | - | - | - | + | + | - | - | - | + | + | - | + | + | - | - | + | - | - | - | n.a. | n.a. | n.a. | - | + | + | + | + | + | - | - | - | + | + | - | - | - | - |
| Celio-Doyle et al. (2008)^8^ | + | + | + | + | + | + | - | - | + | + | + | + | + | - | + | + | + | + | + | - | - | - | - | - | - | - | + | + | - | + | + | + | + | + | - | - | + | + | - | n.a. | - | - | - |
| Cousineau et al. (2010)^9^ | - | + | - | + | + | + | - | - | - | + | + | + | - | - | - | + | + | + | + | - | - | - | - | - | - | n.a. | n.a. | n.a. | - | + | + | + | + | - | + | - | + | - | - | n.a. | - | - | - |
| Franko et al. (2013)^10^ | - | + | - | + | + | + | - | - | - | + | + | + | - | - | + | + | + | + | + | - | + | + | - | - | - | - | - | - | - | + | + | + | + | - | - | - | + | + | - | n.a. | - | - | - |
| Heinicke et al. (2007)^11^ | + | + | + | + | - | + | - | + | - | + | + | + | + | - | - | + | + | + | + | - | - | + | - | - | - | - | + | + | - | + | + | + | + | - | - | - | + | - | + | + | - | - | - |
| Jacobi et al. (2008)^12^ | + | + | + | + | + | + | - | + | + | + | + | + | - | - | + | + | + | + | + | - | + | + | - | - | - | - | + | + | - | + | + | + | - | + | - | - | - | - | + | + | + | - | - |
| Jones et al. (2008)^13^ | + | + | + | + | + | + | - | - | + | + | + | + | + | - | - | + | + | + | + | - | - | + | - | - | - | n.a. | n.a. | n.a. | - | + | + | + | - | + | + | - | - | + | + | + | - | - | - |
| Jones et al. (2012)^14^ | + | + | + | + | + | + | - | - | - | + | + | - | - | - | + | - | + | + | + | - | - | - | - | - | - | - | + | + | - | + | + | + | - | + | - | - | - | + | - | n.a. | - | - | - |
| Jones et al. (2014)^15^ | + | - | - | + | - | + | - | - | - | + | + | + | + | - | + | - | + | + | + | - | + | + | - | - | - | n.a. | n.a. | n.a. | - | + | + | + | + | - | - | - | + | + | - | n.a. | + | - | - |
| Kindermann et al. (2017)^16^ | + | + | - | + | - | + | - | - | - | + | - | - | - | - | - | - | - | + | - | - | - | - | - | - | - | - | - | - | - | + | + | + | - | + | - | - | - | - | - | n.a. | - | - | - |
| Lindenberg & Kordy (2015)^17^ | - | + | + | + | + | + | - | - | + | + | + | + | + | - | + | + | + | + | + | + | + | - | - | - | - | - | - | - | - | + | + | + | + | + | - | - | + | - | + | + | + | - | - |
| Luce et al. (2005)^18^ | + | - | - | + | + | + | - | - | - | + | + | - | - | - | - | - | + | + | + | - | - | + | - | - | - | n.a. | n.a. | n.a. | - | + | - | - | - | - | - | - | - | - | - | n.a. | - | - | - |
| Martinsen et al. (2014)^19^ | - | + | + | + | + | + | - | - | + | + | + | - | - | - | + | + | + | + | + | + | + | + | - | - | - | - | - | - | - | + | + | + | - | + | - | - | - | - | + | + | - | - | - |
| McVey et al. (2009)^20^ | - | + | - | + | - | + | - | - | - | + | + | - | - | - | + | - | + | + | - | - | + | - | - | - | - | - | - | - | - | + | + | + | + | + | - | - | - | + | - | n.a. | - | - | - |
| Minarik et al. (2013)^21^ | + | + | - | + | + | + | - | - | - | + | - | - | - | - | + | - | + | + | + | - | + | - | - | - | - | + | + | + | - | + | + | + | - | + | - | + | - | - | - | n.a. | + | - | + |
| Moessner et al. (2016)^22^ | + | + | - | + | + | - | - | - | - | + | - | - | - | - | - | - | - | + | + | + | + | + | - | - | + | n.a. | n.a. | n.a. | + | + | - | - | - | - | - | - | - | - | - | n.a. | - | - | - |

**Table S4. (continued)**

| **Reference** | **REACH** | | | | | | | | | | **EFFICACY / EFFECTIVENESS** | | | | | | | **ADOPTION** | | | | | | | | | | | | **IMPLEMENTATION** | | | | | | | | | **MAINTENANCE** | | | | |
| --- | --- | --- | --- | --- | --- | --- | --- | --- | --- | --- | --- | --- | --- | --- | --- | --- | --- | --- | --- | --- | --- | --- | --- | --- | --- | --- | --- | --- | --- | --- | --- | --- | --- | --- | --- | --- | --- | --- | --- | --- | --- | --- | --- |
|  | R1 | R2 | R3 | R4 | R5 | R6 | R7 | R8 | R9 | R10 | E1 | E2 | E3 | E4 | E5 | E6 | E7 | A1 | A2 | A3 | A4 | A5 | A6 | A7 | A8 | A9 | A10 | A11 | A12 | I1 | I2 | I3 | I4 | I5 | I6 | I7 | I8 | I9 | M1 | M2 | M3 | M4 | M5 |
| Moessner et al. (2016b)^23^ | + | + | - | + | - | + | - | - | - | + | + | - | - | - | - | - | - | + | + | + | - | - | - | - | - | - | + | + | - | + | + | + | - | + | - | - | - | - | - | n.a. | - | - | - |
| Rodgers et al. (2018)^24^ | - | - | - | + | - | + | - | - | - | + | + | - | - | - | - | + | + | + | + | - | - | + | - | - | - | n.a. | n.a. | n.a. | - | + | + | + | + | - | - | - | + | + | - | n.a. | - | - | - |
| Taylor et al. (2012)^25^ | + | - | - | + | + | - | - | - | - | + | + | - | - | - | + | - | + | + | + | - | + | + | - | - | - | - | + | - | - | + | + | + | + | - | - | - | - | - | - | n.a. | + | - | - |
| Watt et al. (2005)^26^ | - | + | - | + | - | + | - | - | - | - | + | - | - | - | - | - | - | + | - | - | - | - | - | - | - | n.a. | n.a. | n.a. | - | + | + | - | + | - | - | - | - | - | - | n.a. | - | - | - |
| Whittemore et al. (2013)^27^ | - | + | + | + | + | + | - | - | - | + | + | + | - | - | + | + | + | + | + | - | - | - | - | - | - | n.a. | n.a. | n.a. | - | + | + | + | - | + | + | - | + | - | + | + | - | - | - |

+ reported, - not reported, n.a. not applicable

**Table S5. Efficacy of Internet-based interventions for uncontrolled and controlled studies**

| **Uncontrolled studies** | | | |
| --- | --- | --- | --- |
| **Reference** | **Outcome domain** | **Pre-post significance** | **Maintenance to any follow-up assessment** |
| Abascal et al. (2004)^6^ | *Eating disorder symptoms*  EDE-Q  EDI  *Knowledge* | +(S)  +(S)  + | n.a.  n.a.  n.a. |
| Jones et al. (2012)^14^ | *Eating disorder symptoms*  ED risk status, EDE-Q, EDI  *Weight/Shape concerns*  *Self-Esteem*  *Perfectionism*  *% Ideal body weight* | not tested for significance, but overall improvement  not tested for significance, no overall improvement  not tested for significance, but overall improvement  not tested for significance, no overall improvement  not tested for significance, but overall improvement | n.a.  n.a.  n.a.  n.a.  n.a. |
| Jones et al. (2014)^15^ | *BMI*  *Weight/Shape concerns*  *Depression*  *Dietary intake*  Fruit and vegetable consumption  Consumption of soda  *Physical activity and sedentary behaviours*  Physical activity  Watching television  Playing video games | +  +(S)  - (adverse effect in one subgroup)  +  +(S) (but adverse effect in other subgroup)  +(S) (but adverse effect in other subgroup)  +(S) (but adverse effect in other subgroup)  +(S) (but adverse effect in other subgroup) | n.a.  n.a.  n.a.  n.a.  n.a.  n.a.  n.a.  n.a. |
| Luce et al. (2005)^18^ | *Eating disorder symptoms*  EDE-Q  EDI  *Weight/Shape concerns* | +  +(S)  + | n.a.  n.a.  n.a. |
| Moessner et al. (2016b)^23^ | *Help-seeking behavior* | not tested for significance; 9.5% started treatment for ED (24.4% related this to programme participation), 41.1% would utilise treatment if needed (>50% related this to programme participation) | n.a. |
| Taylor et al. (2012)^25^ | *BMI*  *Weight/Shape concerns*  *Dietary intake* (Fruit and vegetable consumption) | +  +  + | n.a.  n.a.  n.a. |
| Watt et al. (2005)^26^ | *Knowledge* | + | n.a. |
| Whittemore et al. (2013)^27^ | *BMI*  *Self-efficacy*  *Sedentary behaviours*  *Dietary intake*  *Physical activity* | -  +  +  +  + | n.a.  yes (overall test of time effects)  yes (overall test of time effects)  yes (overall test of time effects)  yes (overall test of time effects) |
| **Controlled studies** | | | |
| **Reference** | **Outcome domain** | **Significance of group x time interaction (relative improvements of intervention group to control group)** | **Maintenance to any follow-up assessment** |
| Bruning Brown et al. (2004)^7^ | *Eating disorder symptoms*  EDE-Q  EDI  *Weight/Shape concerns*  *Knowledge*  *Parental Attitudes and criticism* | +  -  -  +  + | no  n.a.  n.a.  n.a. (not assessed at FU)  n.a. (not assessed at FU) |
| Celio-Doyle et al. (2008)^8^ | *Eating disorder symptoms* (EDE-Q)  *BMI*  *Use of eating-related and physical activity skills* | +  +  + | no  no  yes |
| Cousineau et al. (2010)^9^ | *Body esteem*  *Self-esteem*  *Knowledge* | +(S)  +(S)  - | yes (overall test of time effects)  yes (overall test of time effects)  n.a. |
| Franko et al. (2013)^10^ | *Body Image*  Body esteem  Body dissatisfaction  Physical appearance comparison | +(S)  +(S)  +(S) | no  no  no |
| Heinicke et al. (2007)^11^ | *Eating disorder symptoms*  DBEQ-Restraint  EDI-Bulimia Extreme weight loss behaviours  *Weight/Shape concerns & Body image*  Body shape questionnaire  Body comparison  Sociocultural attitudes towards appearance  *BMI*  *Depression* | +  +  +  +  +  +  -  + | yes  yes  yes  yes  yes  yes  n.a.  yes |
| Jacobi et al. (2018)^12^ | *Eating disorder symptoms*  *EDE Interview (any scale)*  *EDI (any scale)*  *Weight/shape concerns*  *BMI (% expected body weight)*  *Excessive exercise* | -  -  -  +  - | n.a.  n.a.  n.a.  yes  n.a. |
| Jones et al. (2008)^13^ | *Eating disorder symptoms*  Objective / subjective binge eating  *Weight/Shape concerns*  *BMI*  *Dietary intake*  *Depression* | +  +  +  -  - | yes  yes  yes  n.a.  n.a. |
| Lindenberg & Kordy (2015)^17^ | *Eating disorder onset* | +(S) | n.a. (incidence of 48 weeks) |
| Martinsen et al. (2014)^19^ | *Eating disorder onset*  *Eating disorder symptoms*  Symptoms (general)  Dieting practices  Unhealthy weight control practices  EDI  *Body dissatisfaction*  *Self-Esteem* | +(S)  +(S)  +(S)  -  -  -  - | yes  no  yes  n.a.  n.a.  n.a.  n.a. |
| McVey et al. (2009)^20^ | *Knowledge*  about physical changes with puberty  about facts concerning restrictive eating  about peer and adult influences  about influences of the media on weight loss  *Efficacy to fight weight bias* | -  +(S)  +(S)  +(S)  +(S) | n.a.  n.a.  n.a.  n.a.  n.a. |
| Rodgers et al. (2018)^24^ | *Body image*  Body esteem  Body Image Acceptance and Action  Physical appearance comparison  *Self-compassion*  *Mood* | +  -  -  +  - | yes (overall test of time effects)  n.a.  n.a.  yes (overall test of time effects)  n.a. |

Abbreviations: + significant, +(S) significant in specific subgroups / subpopulations only, - not significant, n.a. not applicable, EDE-Q Eating Disorder Examination Questionnaire, EDI Eating Disorder Inventory, BMI Body Mass Index, DBEQ Dutch Behavior Eating Disorder Questionnaire

**Table S6. Adherence and compliance measures reported in included studies**

| **Reference** | **Number / percentage of lessons completed** | **Frequency of logins / Length of use** | **Self-monitoring journals** | **discussion forum** | **chat use** | **Other** |
| --- | --- | --- | --- | --- | --- | --- |
| Abascal et al. (2004)^6^ |  |  | ø 2.5-15.2 (dependent on group and type of journal) | ø 6.9-9.7 forum entries (dependent on group) |  |  |
| Bruning Brown et al. (2004)^7^ | 73% of parents read > 80% of content |  |  | total number of postings: 9 |  |  |
| Celio-Doyle et al. (2008)^8^ | ø 29.9% of material viewed, 35% viewed < 10% of screens |  |  |  |  |  |
| Jacobi et al. (2018)^12^ | ø 2.7 of 6 programme sessions opened (median: 2.0)  ø percentage of programme pages opened: 28% /median: 16%)  16% opened more than 75% of programme pages | ø 3.4 logins (range: 0-11, median: 3.0)  29% never logged on |  |  |  |  |
| Jones et al. (2008)^13^ | 27% used some programme components ≥ 8 weeks, 42% for 1-7 weeks  ø 19.4% of content screens viewed |  | ø 21.4 | ø 5.2 forum postings |  | ø 1 mentor session attended |
| Jones et al. (2012)^14^ | 39.1% (total) viewed all sessions  ø 1.5-5.1 of 8 sessions viewed (dependent on sample) |  | ø 0-4 (dependent on type of journal and sample) | ø 0-2.7 (dependent on sample) |  |  |
| Kindermann et al. (2017)^16^ | ø 154.9 number of page visits (range:0-9133) | ø 6.2 (range 2-52) monthly periods | 19.4% used monitoring (but not programme)  ø 13.1 (range 2-197) | ø 82.8 (range 0-6357 forum visits  ø 3.0 (range 0-475) forum post  13.2% ≥ 1 post | 17% used individual or group chats (range: 0-65) |  |
| Lindenberg & Kordy (2015)^17^ | 98.6-98.8% used website (dependent on wave) |  | 74.6%-76.4% used monitoring (dependent on wave) | 77%-81% used forum (dependent on wave) |  | 0.7%-2.6% used optional face-to-face counselling (dependent on wave) |

**Table S6 (continued)**

| **Reference** | **Number / percentage of lessons completed** | **Frequency of logins / Length of use** | **Self-monitoring journals** | **discussion forum** | **chat use** | **Other** |
| --- | --- | --- | --- | --- | --- | --- |
| Martinsen et al. (2014)^19^ | 89.9% attended ≥ 3 of 4 sessions (55.5% attended all)  69.4% of coaches attended seminar |  | 28.3% used diary |  |  | 94.3% joined Facebook group  100% completed practical assignments, 80.2% completed theoretical assignments |
| McVey et al. (2009)^20^ |  |  |  |  |  | Teachers’ use of modules in classroom: 91% used ≥ 1 module, 45.5% used all modules |
| Minarik et al. (2013)^21^ |  | ø 6 (40% one login, 66% 1-5 logins, 11% > 5 logins |  | 5% used forum, 13367 total visits | total group chats: 146, total individual chats: 100 |  |
| Moessner et al. (2016b)^23^ |  | ø 3.07-13.27 (dependent on group) | ø 2.24-5.07 (based on groups) | ø 4.78-19.01 forum visits  ø 0.37-1.00 forum entries  (dependent on group) | ø 0.09-0.44 (dependent on group) |  |
| Whittemore et al. (2013)^27^ | ø 83%, median: 100% |  | ø 5.3, median: 5 |  |  |  |

**References:**

1. O’Connell ME, Boat T, Warner KE. Defining the Scope of Prevention [Internet]. National Academies Press (US); 2009 [cited 2018 Jul 16]. Available from: https://www.ncbi.nlm.nih.gov/books/NBK32789/

2. Proudfoot J, Klein B, Andersson G, Carlbring P, Kyrios M, Munro C, et al. Guided CBT internet interventions: Specific issues in supporting clients with depression, anxiety and comorbid conditions. In: Bennett-Levy J, ORCID: 0000-0003-0998-116X; Richards DA, ; Farrand P, ; Christensen H, ; Griffiths KM, ; Kavanaugh DJ, et al., editors. Oxford guide to low intensity CBT interventions. Oxford University Press; US; 2010. p. 253–64.

3. Erbe D, Eichert H-C, Riper H, Ebert DD. Blending Face-to-Face and Internet-Based Interventions for the Treatment of Mental Disorders in Adults: Systematic Review. J Med Internet Res [Internet]. 2017 Sep 15 [cited 2019 May 20];19(9). Available from: https://www.ncbi.nlm.nih.gov/pmc/articles/PMC5622288/

4. Juarascio AS, Manasse SM, Goldstein SP, Forman EM, Butryn ML. Review of Smartphone Applications for the Treatment of Eating Disorders. Eur Eat Disord Rev. 2015;23(1):1–11.

5. Fridrici M, Lohaus A, Glaß C. Effects of incentives in web-based prevention for adolescents: Results of an exploratory field study. Psychol Health. 2009 Jul 1;24(6):663–75.

6. Abascal L, Bruning Brown J, Winzelberg AJ, Dev P, Taylor CB. Combining universal and targeted prevention for school-based eating disorder programs. Int J Eat Disord. 2004 Jan;35(1):1–9.

7. Bruning Brown J, Winzelberg AJ, Abascal LB, Taylor CB. An evaluation of an Internet-delivered eating disorder prevention program for adolescents and their parents. J Adolesc Health. 2004;35(4):290–6.

8. Celio Doyle AA, Goldschmidt A, Huang C, Winzelberg AJ, Taylor CB, Wilfley DE. Reduction of overweight and eating disorder symptoms via the Internet in adolescents: a randomized controlled trial. J Adolesc Health Off Publ Soc Adolesc Med. 2008 Aug;43(2):172–9.

9. Cousineau TM, Franko DL, Trant M, Rancourt D, Ainscough J, Chaudhuri A, et al. Teaching adolescents about changing bodies: Randomized controlled trial of an Internet puberty education and body dissatisfaction prevention program. Body Image. 2010 Sep;7(4):296–300.

10. Franko DL, Cousineau TM, Rodgers RF, Roehrig JP. BodiMojo: effective Internet-based promotion of positive body image in adolescent girls. Body Image. 2013 Sep;10(4):481–8.

11. Heinicke BE, Paxton SJ, McLean SA, Wertheim EH. Internet-delivered targeted group intervention for body dissatisfaction and disordered eating in adolescent girls: a randomized controlled trial. J Abnorm Child Psychol. 2007 Jun;35(3):379–91.

12. Jacobi C, Hütter K, Völker U, Möbius K, Richter R, Trockel M, et al. Efficacy of a Parent-Based, Indicated Prevention for Anorexia Nervosa: Randomized Controlled Trial. J Med Internet Res. 2018 Dec 14;20(12):e296.

13. Jones M, Luce KH, Osborne MI, Taylor K, Cunning D, Doyle AC, et al. Randomized, controlled trial of an internet-facilitated intervention for reducing binge eating and overweight in adolescents. Pediatrics. 2008 Mar;121(3):453–62.

14. Jones M, Völker U, Lock J, Taylor CB, Jacobi C. Family-based early intervention for anorexia nervosa. Eur Eat Disord Rev J Eat Disord Assoc. 2012 May;20(3):e137-143.

15. Jones M, Taylor Lynch K, Kass AE, Burrows A, Williams J, Wilfley DE, et al. Healthy weight regulation and eating disorder prevention in high school students: a universal and targeted Web-based intervention. J Med Internet Res. 2014 Feb 27;16(2):e57.

16. Kindermann S, Moessner M, Ozer F, Bauer S. Associations between eating disorder related symptoms and participants’ utilization of an individualized Internet-based prevention and early intervention program. Int J Eat Disord. 2017 Oct;50(10):1215–21.

17. Lindenberg K, Kordy H. Efficacy of an internet-delivered tiered strategy for eating disorder prevention in high school students. [German]. Kindh Entwickl Z Klin Kinderpsychol. 2015;24(1):55–63.

18. Luce KH, Osborne MI, Winzelberg AJ, Das S, Abascal LB, Celio AA, et al. Application of an algorithm-driven protocol to simultaneously provide universal and targeted prevention programs. Int J Eat Disord. 2005 Apr;37(3):220–6.

19. Martinsen M, Bahr R, Børresen R, Holme I, Pensgaard AM, Sundgot-Borgen J. Preventing eating disorders among young elite athletes: a randomized controlled trial. Med Sci Sports Exerc. 2014 Mar;46(3):435–47.

20. McVey G, Gusella J, Tweed S, Ferrari M. A controlled evaluation of web-based training for teachers and public health practitioners on the prevention of eating disorders. Eat Disord. 2009 Feb;17(1):1–26.

21. Minarik C, Moessner M, Ozer F, Bauer S. [Implementation and dissemination of an internet-based program for prevention and early intervention in eating disorders]. Psychiatr Prax. 2013 Sep;40(6):332–8.

22. Moessner M, Minarik C, Ozer F, Bauer S. Effectiveness and Cost-effectiveness of School-based Dissemination Strategies of an Internet-based Program for the Prevention and Early Intervention in Eating Disorders: A Randomized Trial. Prev Sci. 2016 Apr 1;17(3):306–13.

23. Moessner M, Minarik C, Özer F, Bauer S. Can an internet-based program for the prevention and early intervention in eating disorders facilitate access to conventional professional healthcare? J Ment Health. 2016 Sep 2;25(5):441–7.

24. Rodgers RF, Donovan E, Cousineau T, Yates K, McGowan K, Cook E, et al. BodiMojo: Efficacy of a Mobile-Based Intervention in Improving Body Image and Self-Compassion among Adolescents. J Youth Adolesc. 2018 Jul 18;47(7):1363–1372.

25. Taylor CB, Taylor K, Jones M, Shorter A, Yee M, Genkin B, et al. Obesity prevention in defined (high school) populations. Int J Obes Suppl. 2012 Jul;2:S30–2.

26. Watt M, Rancourt D, Cousineau TM, Franko DL. A Pilot Study of a Multimedia Puberty Module for Middle Schoolers. J Nutr Educ Behav. 2005;37(4):211–2.

27. Whittemore R, Jeon S, Grey M. An Internet Obesity Prevention Program for Adolescents. J Adolesc Health. 2013 Apr;52(4):439–47.
